# ‘*It was something that I let slip by the wayside*’: a qualitative study of challenges to equitable mpox vaccination in the UK outbreak response

**DOI:** 10.1186/s12889-025-24694-w

**Published:** 2025-10-31

**Authors:** Chloé Pasin, Benjamin Weil, Rosalie Hayes, Qaisar Siddiqui, Isabelle Whelan, Will Nutland, Ismael Maatouk, Chloe M Orkin, Sara Paparini

**Affiliations:** 1 SHARE Collaborative, Blizard Institute, Queen Mary University of London, London, UK; 2 The Love Tank CIC, London, UK; 3 SHARE Collaborative, Wolfson Institute of Population Health, Queen Mary University of London, London, UK; 4 Centre for Public Health and Policy, Wolfson Institute of Population Health, Queen Mary University of London, London, UK; 5 Global HIV, Hepatitis and Sexually Transmitted Infections Programmes, World Health Organization, Geneva, Switzerland; 6 Barts Health NHS Trust, London, UK

**Keywords:** Mpox, Vaccination, Equity, Health inequalities, Infectious diseases

## Abstract

**Background:**

During the mpox outbreak in 2022-2023, inequities in vaccine access and uptake were highlighted in several countries. We present an intersectionality-informed, equity-focused approach to understand how the intersection of several characteristics, practices, and circumstances created unique and specific barriers to accessing the mpox vaccine in the UK.

**Methods:**

The study was co-produced with community co-researchers. Between April and July 2023, semi-structured in-depth interviews and focus group discussions were conducted online with 35 UK-based people of diverse ethnicity, sexual identity and orientation with risk factors for mpox. Interviews and focus groups were audio-recorded and transcribed. Transcripts and codes were analysed applying an equity lens focused on understanding how the intersection of demographics, practices and circumstances might lead to exclusion from the mpox vaccination public health campaign. Combining data from all participants, we designed four composite fictional *personas* who correspond to representative profiles of people that found themselves at the margins of the UK mpox vaccination campaign.

**Results:**

The thirty-five participants were diverse in terms of gender (28 cisgender men, 7 transgender or non-binary) and ethnicity (including White, Black, Asian, Latino/x and Arab individuals). We identified six circumstances leading to the exclusion of the UK mpox vaccination campaign: i) not being connected to sexual health services or community organisations and advocates; ii) feeling that the vaccine criteria are too restrictive; iii) lacking trust in institution, often because of structural racism; iv) experiencing feasibility barriers; v) living outside of London; vi) being in a relationship with someone with more risk factors. Our four fictional personas combine these circumstances and represent the characteristics of someone on the margins, either geographically or socially.

**Conclusion:**

Our findings highlight the need to design more inclusive vaccine campaigns during evolving public health emergencies that consider how the intersection of stigma, characteristics, practices, and circumstances might lead to exclusion and inhibit vaccine uptake. Our persona approach further highlights the need to consider whether shared barriers to engagement (which can span multiple identities and positionalities) require tailored solutions. Guidelines should be co-designed with affected communities and include stigma-reduction interventions to ensure equitable provision and access to vaccine programmes.

**Supplementary Information:**

The online version contains supplementary material available at 10.1186/s12889-025-24694-w.

## Introduction

Mpox has been a long-standing, under-resourced, under-researched and neglected infection in Central and Western Africa. As a result of this neglect, two overlapping global outbreaks have become urgent and ongoing priorities for global public health policy since 2022. This has necessitated the declaration of two public health emergencies of international concern (PHEIC) by the World Health Organization (WHO), first in 2022–2023 during the multi-country Clade IIb mpox virus (MPXV) outbreak, then in 2024 (an ongoing PHEIC) following the emergence of a new MPXV sub-clade (designated as Ib) [1–5]. The UK has been largely affected by the clade IIb outbreak, with more than 3700 recorded cases [6]. In the UK the epidemiological pattern mirrored that of other countries in the multi-country outbreak, in that gay, bisexual, and other men who have sex with men (GBMSM) and their sexual partners were predominantly affected. People living with HIV made up close to 50% of people affected during the first months of the 2022 outbreak [7, 8]. During the Clade IIb outbreak, person-to-person mpox transmission occurred predominantly through close sexual contact [9]. Sexual activity has driven transmission within GBMSM sexual networks [8, 10].

For these reasons, the multi-agency public health response in the UK led by UK Health Security Agency (UKHSA), together with the sexual health clinicians within the National Health Service (NHS), community organisations, and professional medical associations, was mostly tailored for GBMSM. Public health interventions consisted of early diagnostic testing for suspected cases, self-isolation for confirmed cases, contact tracing, and the promotion of harm reduction strategies, such as reducing the number of sexual partners and health awareness around symptom recognition [11]. In addition, a reactive vaccination campaign was initiated by UKHSA in June 2022, using the MVA-BN vaccine originally developed for smallpox prevention and manufactured by Bavarian Nordic, and subsequently authorised for emergency use to prevent mpox [12]. Initially, shortages of the vaccine limited availability to a single dose until further supplies became available, and were later also delivered intra-dermally in smaller doses to stretch vaccination capacity [13]. Many sexual health services opted to deliver ‘mass vaccination events’ in many cases using existing infrastructure from COVID-19 vaccination centres to provide vaccines to people who needed vaccine protection on a ‘first-come, first-served’ basis [13]. These mass vaccination events often involved queuing visibly in the street to access the building, which is likely to deter GBMSM from those communities where significant interpersonal and societal stigma around LGBTQ + identities persists.

Community organisations and local sexual health services played a critical role in the vaccination roll-out, building on community trust, peer-to-peer education, and through social media campaigns [14–17]. Online surveys conducted with GBMSM before, during, and after the vaccination campaign have shown high mpox vaccine acceptability and uptake [18–28]. However, these surveys also highlighted barriers to vaccination, including low risk perception, fear of sexual stigma, and limited access to sexual health clinics [21–23, 29, 30]. In a survey conducted in the UK before the vaccination campaign, respondents identifying as being Black and Other heritage groups reported a lower intention to take up the vaccine than people of White heritage groups [18]. Acceptability and uptake of mpox vaccines were found to be lower among people self-identifying as bisexual or heterosexual, reporting lower educational qualifications, living in rural areas, or belonging to a racially minoritised group [18–20, 31–33]. A few, limited qualitative studies with GBMSM (including migrants) have provided further insights into practical issues around vaccine access, stigma around mpox, and the crucial role of social media for information sharing during the mpox outbreak in the UK [15, 34–37]. Overall, the mpox vaccine literature in the UK (and in similar settings such as the US) highlights inequities in vaccine access and uptake and the potential for further exacerbation of these inequities in future outbreak responses if they are not taken into consideration in public health guidelines [38–41].

Previous work has mostly focused on identifying *specific demographic characteristics (e.g. ethnicity; gender)* and commenting on the relevance of these characteristics for the equitable implementation of vaccination [42, 43]. However, to understand the full picture and design inclusive public health guidance it is important to consider how different social determinants of health inequalities come together to produce different and unequal experiences of public health responses to infectious disease outbreaks. The intersection of several characteristics (e.g. gender, ethnic background), practices (e.g. kinds of sex) and circumstances (e.g. living arrangements) creates unique and specific disadvantages in the context of accessing the mpox vaccine, which also have broader relevance for other public health responses to infectious diseases. We thus present here an intersectionality-informed [44], equity-focused approach to understanding different experiences of people affected by the mpox outbreak in the UK in 2022–2023 as narrated in our qualitative study. Further, we contextualise our findings against dilemmas regarding decisions made during outbreaks, such as promoting vaccine accessibility versus rationing, or tailored vaccination campaigns versus general communication.

## Methods

This exploratory qualitative study was part of a commission by WHO Health Emergencies Programme focusing on understanding the values and preferences of most affected communities in relation to preventive strategies against mpox [45]. The study was codesigned and delivered by the SHARE Research Collaborative for Health Equity, based at Queen Mary University of London (QMUL), and The Love Tank, a non-profit at the forefront of the mpox community response in the UK. All data collection materials were co-created between the university and community partners. Recruitment and data collection were conducted by The Love Tank researchers. Analysis was done in collaboration.

Between April and July 2023, semi-structured in-depth interviews and focus group discussions were conducted online with people living in the UK with risk factors for mpox. At that time, the UK mpox outbreak was mostly controlled, with less than 5 confirmed mpox cases per week [46] and vaccination efforts were continuing for both those receiving their first dose and those receiving their second one. The spike of cases had occurred in summer 2022, so the accounts were relatively fresh in participants’ mind. Recruitment was conducted using Love Tank’s existing networks and via social media. To be eligible for the study, participants had to be either a person who has sex with men who have sex with men (e.g., GBMSM, women who have sex with bisexual men, or non-binary people who have sex with men) or to have experienced mpox. They were also required to be aged 18 or above, currently living in the UK, and to have lived in the UK during the 2022 mpox outbreak. Identity markers, sexual practices and geographic information were used to identify suitable interview participants.

### Data collection

Focus groups were conducted to explore community-level attitudes towards mpox prevention interventions [47, 48], while in-depth interviews were conducted to encourage participants to discuss any individual behaviour change in conditions that would minimise the fear of potential judgement from peers [47]. All participants were reimbursed for their time. We developed topic guides for both the focus groups and interviews (see supplementary material).

### Data analysis

Interviews and focus groups were audio-recorded and transcribed. We analysed them using framework analysis, a structured method for analysing and interpreting qualitative data by classifying it and organising it by themes through a coding process which follows a deductive approach (looking out for specific topics in the data) alongside an inductive one (allowing data to lead to review and modification of the original topics) [49]. Vaccination emerged as a key topic during primary coding. Subsequently, transcripts and codes were reviewed by CP and RH applying an ‘equity lens’, that is, an intentional focus on understanding how the intersection of *demographics*,* practices* and *circumstances* might simultaneously shape mpox risk but also lead to exclusion from a public heath intervention (here the mpox vaccine) for members of most-affected communities.

We present our results by designing four composite *personas*: fictional persons written from actual data [50–53] who correspond to representative profiles of people that found themselves at the margins of the UK mpox vaccination campaign. The choice of this method of analysis and presentation of findings was based on our aim to better reflect the diverse, intersectional needs of people during the vaccination campaign to offer a comprehensive view of inclusion challenges during the public health response. Using personas as a narrative device, we wish to provide concrete tools to guide policy and decision makers. Composite narratives have been suggested in the literature as ways to engage the reader with the experiences of the participants and to present the complexities of people’s lives in an accessible way, which is potentially useful in contexts outside academia (e.g., health communication, community, policy) [52–55].

### Creation of personas

We used six principles to create the personas: (i) *relatability*: we ensured that our personas were relatable to the community co-authors based on their experiences of working in the field and in community with underserved and marginalised groups and their in-depth knowledge of participants’ accounts through the qualitative data collection we conducted together. We aimed to emphasise that people with different identities and positionalities can share similar experiences (i.e. that experience is not solely reducible to identity); (ii) *credibility*: although the different personas are constructed to represent the intersection of different characteristics, we reflected intersections that were identified and grounded in the data; (iii) *equity*: we aimed to reflect how needs of marginalised populations might differ; (iv) *frequency*: if some barriers to accessing the vaccine were mentioned often in the data, we aimed at including them in different personas to show how these barriers could be shared among people at different intersections; (v) *novelty*: we included barriers and circumstances that have not – to our knowledge - been previously fully described in the literature: (vi) *reflexivity*: we conducted regular meetings to reflect on the process of creation of personas and discuss potential biases related to our positionalities as researchers that could emerge in their creation.

We acknowledge that by creating personas, we are not able to represent all circumstances and intersections leading to the exclusion from the mpox vaccination campaign. Our approach strikes a balance between representativeness and relevance. By following these six principles, we aim to ensure that the personas are read through the lens of practices and *circumstances* and not only identities or demographic characteristics, as it is more often the case in the literature.

The following steps were taken to create the personas: first, we coded the transcripts using vaccine-related themes (e.g., fear and anxiety, lack of information, perceived lack of government response, difficulty accessing the vaccine) [15, 35]. Second, using an equity lens, we further identified populations at unique risk for inequities and circumstances leading to exclusion from the vaccination campaign. As a third step, we then combined at least two circumstances in each persona and attributed the corresponding characteristics to that persona. If applicable to more than one group, some circumstances were used more than once. Finally, to provide a more complete picture of each persona, we included additional themes that emerged from our initial coding and accounted for a potential timeline effect (e.g., the effect of the COVID-19 pandemic, limited information at the beginning of the mpox outbreak). CP, RH, and SP drafted the personas. BW reviewed and commented on them, helping to re-frame how personas were constructed and used, including regarding the intersectionality and relatability aspects.

Anonymised quotes from interview and focus group transcripts are used to illustrate the narratives in the study. The quotes are verbatim and derived from individual interviews with multiple participants but are woven into a single persona.

### Findings

#### Study population

There were 35 participants in total. An initial round of recruitment invited participants to take part in a focus group. Participants could choose either to attend a focus group for anyone who is eligible (*n* = 5), or to take part in a focus group exclusively for people who had been diagnosed with or believed that they had had mpox (*n* = 5) – to minimise the fear of potential judgement from peers. A second round of recruitment invited participants (*n* = 22) to take part in in-depth interviews. A final round of recruitment invited participants (*n* = 3) to take part in a final focus group for eligible participants. Table 1 outlines self-reported participant characteristics.

**Table 1:** Self-reported participant characteristics

Characteristic	*n* (%)
*Gender identity*	
Cisgender man	28 (80)
Genderqueer/non-binary person	4 (11)
Transgender man	2 (6)
Transgender woman	1 (3)
*Assigned sex at birth*	
Male	33 (94)
Female	2 (6)
*Sexual orientation*	
Gay	27 (77)
Queer	5 (14)
Bisexual	1 (3)
Pansexual	1 (3)
Dyke	1 (3)
*Ethnicity*	
White British/European/Other	23 (66)
Black British/African/Caribbean	4 (11)
Latino/Latinx	3 (9)
Asian British/Pakistani/Indian/Other	3 (9)
Arab	2 (6)
*Disability*	
No disabilities	27 (77)
Has a disability or long-term health condition	7 (20)
Prefer not to say	1 (3)
*Religion*	
No religion	20 (57)
Christian	9 (26)
Muslim	2 (6)
Agnostic	2 (6)
Spiritual	1 (3)
Sikh/Buddhist^1^	1 (3)
*Region of birth*	
Europe^2^	25 (71)
Latin America	3 (9)
Africa	2 (6)
Middle East	2 (6)
Caribbean	1 (3)
Australasia	1 (3)
Prefer not to say	1 (3)
*Immigration status*	
UK citizen	20 (57)
UK resident (various forms)	13 (37)
Student visa	1 (3)
Asylum seeker	1 (3)
*Employment status*	
Full-time employed	21 (60)
Self-employed	7 (20)
Part-time employed	4 (11)
Student	2 (6)
Unemployed	1 (3)
	Median (IQR)
*Age*	31 (29, 36)

^1^ One participant identified as both Sikh and Buddhist

^2^ 18 of the 25 participants born in Europe were born in the United Kingdom

#### Circumstances leading to exclusion from the vaccination campaign

Our analysis identified the following six circumstances leading to exclusion from the vaccination campaign, some applying to different groups and identities: (i) not being connected to sexual health services or community organisations and advocates (on social media for example); (ii) feeling that the criteria for accessing the vaccine are too restrictive (e.g., don’t apply to their body, sexual practices); (iii) lacking trust in institution, often because of structural discrimination (e.g., racism); (iv) experiencing feasibility barriers to accessing the vaccine (e.g., not being able to navigate the administrative system, not having time to stay in line); (v) living outside of London; (vi) being in a relationship with someone with more risk factors. These circumstances are reflected in the following personas.

#### Personas

The following personas combine mpox vaccination experience of multiple participants but are written as a fictional individual experiences.

##### Persona 1. John

John is a White, cisgender, gay man, living in a rural area in the south of England. He travels to London occasionally to meet friends and attend social events. He is single and regularly uses social ‘hook-up’ apps to meet sexual partners. He is conscious of the need to regularly screen for STIs and HIV, but the nearest sexual health service is far from where he lives and doesn’t offer home testing, so he feels like he doesn’t get tested as often as he should. This is made more challenging by his ADHD, which sometimes affects his ability to plan and organise his time:

*“I feel very strongly that while obviously even in London the information and the services are inadequate*,* if you are LGBT + outside of London*,* it becomes much harder. (…) Like (…) if I was to say*,* put my address as being somewhere in London*,* I can go to Dean Street* [sexual health clinic in central London] *and get like a test in like 24 hours a lot*,* which to me is insane. Like in* [where John lives], *you have to*,* it’s so hard to get to get a sexual health checkup. And it’s very long and you’ve got to travel quite far to the place*,* and there’s only one place that does it”. (Q5)*

During the mpox outbreak, John was mostly getting information about mpox through social media. He followed several social media accounts of sexual health charities and gay activists to help him stay up to date. When the vaccine roll-out began, he saw advice being shared about how to access it – but mostly this related to London clinics. Some local friends travelled to London to obtain the vaccine either at a sexual health clinic (“*everyone kind of had travel all over London trying to find like the last few spots (Q2)”)* or at community events where he remembers “*them traveling to London to attend UK Black Pride* [a music and culture festival] *to get it” (FG2*,* S5).*

At that time, John couldn’t to travel to London, as he had lost all his freelance work during the COVID-19 pandemic and was worried about finances. He tried to find out if he could get vaccinated at his local clinic, but there was very limited information online and whenever he tried to call, he couldn’t get through to anyone. After a couple of attempts, he gave up:

*“However*,* actually obtaining a vaccination for mpox from memory was quite difficult. It wasn’t quite as readily available as the COVID vaccines were. I remember seeing quite frequently*,* again on Twitter. People quite literally just posting you know*,* get yourself to this hospital now because they’ve got a surplus of vaccinations and it’s ‘first come first serve’. Now*,* obviously*,* that’s not convenient for everyone*,* which is why I never actually got vaccinated because*,* really what’s the word I’m looking for? While I knew it was a potentially*,* um going to impact me or could have potentially impacted me. I feel like the distribution or vaccinations wasn’t quite as well executed or prioritized in the same way things like COVID was. So you know*,* if all the information I’m getting is saying you need to get vaccinated*,* but you can only get vaccinated on an ad hoc basis when it’s not really convenient to you*,* then it was something that I let slip by the wayside”. (Q3)*

Reflecting on this later, he noted that there was a stark difference between the mpox and COVID-19 vaccination campaigns, which left him feeling that LGBTQ + health is less of a priority for the government:

*“I don’t trust the government in power at the moment at all*,* particularly when it comes to minority rights” (B5).*

##### Persona 2. Amir

Amir is a 19-year-old cisgender gay man of Pakistani heritage, living in Birmingham. Amir has struggled with his sexual identity for several years and has only recently started identifying as gay. He is worried about how his family or friends might respond to this, so he avoids following any accounts on social media that could ‘out’ him (e.g., LGBTQ + activists, sexual health services oriented towards GBMSM, etc.). He tends to meet men at cruising hotspots on the other side of the city, and although he tries to use condoms, this doesn’t always happen.

At the beginning of the outbreak, Amir saw an item about mpox on the BBC News. When he saw that gay men were considered at high risk, he panicked and started googling symptoms. The extreme visible mpox symptoms he found circulating online made him feel very anxious. But he also felt annoyed that the media and guidance was focusing on gay men specifically, as he felt this made it more difficult for him to get tested or treated without raising suspicions about his sexuality amongst his peers:

*“And it looks like it was exclusively for the gay people. And initially*,* even the press was kind of targeting just homosexuals for reasons like*,* you know*,* perhaps promiscuity*,* or different approach to sex perhaps”.* (B4)

Amir was aware that there was a vaccine but wasn’t sure about whether he wanted it or not. On the one hand, he was worried about his risk of getting mpox and the consequences of this, but on the other, he was a little suspicious of potential side-effects and whether it would even work – since he’d read that the vaccine wasn’t even originally designed for mpox:

*“I would say there is no other choices*,* you know*,* it was either you get it*,* you get it or you don’t*,* you know*,* (…) I don’t know what’s inside that that liquid*,* you know*,* at the end of the day*,* and and you’re just gonna take risk*,* it’s even you you’re gonna risk your life or you follow what’s the guidelines is all about*,* you know*,* and and*,* yeah*,* and try to protect yourself. (…) If you don’t like I don’t know*,* if injecting something that is going to affect me in the long run*,* you know*,* because there were some side effects for people who got injections where they get some part of that some liver*,* lung issues and all of that. So*,* you know*,* it was option A or B*,* at the end of the day”. (B6)*

Since the only way he was going to get it was to queue for hours outside a sexual health clinic with other gay men (“*It was obviously an MSM* [men who have sex with men] *clinic on a Wednesday night” (B5))*, he decided it just wasn’t worth it.

##### Persona 3. Luka

Luka was granted refugee status on the basis of his sexual orientation in early 2022. He lives in London and has embraced the queer scene there – where he feels able to live his life openly as a gay (cisgender) man. He has been in a relationship with his partner Luis since 2022 – Luis is a sex worker producing digital adult film content. Luka and Luis are romantically monogamous, and Luka doesn’t have sex with anyone other than Luis.

When the mpox outbreak began, Luka wasn’t especially worried about getting mpox. Although he was conscious that Luis’ work exposed him to potentially greater risk of acquiring mpox, he also knew that Luis is very attentive to his sexual health, because he can’t afford to miss work due to infection or sickness:

*“So I still felt like I could potentially be at risk*,* but I didn’t feel like I was that high of a risk*,* which is why I didn’t necessarily go to get the vaccine when it first came out because I was like*,* Well*,* I’m not going out and I’m monogamous and yeah” (B8).*

When the vaccine became available, Luis got vaccinated as soon as he could, but Luka didn’t feel he would fit the eligibility criteria – “*the stringent criteria in in which we kind of meet or you have to have this*,* you had to be having sex with this amount of people*” *(FG2 S2)*. Moreover, Luka is conscious about taking a finite resource – “I *think I understand there not being enough to cover everybody. And I understand from that from the public health perspective where let’s cover the persons who will be the most at risk”* (*FG2 S2)* – and impacting access for others who Luka thinks need it more (“*like better that other people get the vaccine first who are more high risk” (B8)*).

Luka had planned to attend Brighton Pride and realised two weeks before the event that there could be a risk for mpox transmission there, due to skin-to-skin contact. He felt that getting vaccinated would help him feel safe and fully enjoy the event. However, he couldn’t find information on where to get vaccinated without an NHS number:

*“So I think for my community of friends*,* because we’re all migrants who moved from home to the UK*,* or kind of based all around the UK […]*,* there was a real sense of panic around like not being able to access the vaccine in certain places and not having the correct kind of credentials*,* I think like*,* my friend didn’t have an NHS number*,* so was harder to find places where they could get the vaccine” (FG2*,* S5).*

##### Persona 4. Jody

Jody is a polyamorous, bisexual, transgender woman, whose partners include another transgender woman and a cisgender bisexual man. She is very open in discussing sexual health within her relationships, and feels a sense of responsibility towards protecting her partners’ health:

*“I went through kind of pretty serious personal losses during COVID. And like this heightened sensitivity to how my actions could affect other people is very much in my consciousness*,* then as it is now*,* you know*,* always with a kind of queer sense of responsibility*,* but really high off the back of the pandemic”. (B5)*

At the beginning of the mpox outbreak, she felt conscious that she might have potentially higher risk of acquiring mpox. However, she found it difficult to source accurate information which took into account her sexuality, her body type, and her gender identity. For example, she couldn’t find any information on what vulval symptoms of mpox might look like, or any advice for people who have had vaginoplasties. She was able to get some advice from transgender friends working in community sexual health, but felt that many transgender people wouldn’t have these same connections.:

*“Lots of the guidance was pretty complicated. And didn’t put to like*,* my specific community very well*,* which was like quite frustrating and like going forward*,* like that there being like stuff that is responsive to the needs of more specific groups*,* would be really good for the next time something happened. And as ever*,* coming to that for trans lesbians*,* like*,* there was very little information about like*,* whether T4T* [trans for trans] *like poly like relationships are likely to be higher risk*,* even though I assume we are going to be much higher risk because of the level of polyamory in the community”. (Q2)*

When the mpox vaccine roll-out began, Jody felt torn about whether to try and access it. She wanted to reduce her risk of acquiring mpox and protect her partners, but she was also a little concerned about how the vaccine might affect her hormone therapy. She wasn’t even sure if she would be eligible since the eligibility criteria seemed targeted to cisgender GBMSM. Ultimately the prospect of having to queue for hours for a vaccine that she may be turned down for didn’t seem worth it:

*“I think just reiterating that point when the vaccines were coming out and it was very much aimed at kind of like*,* gay or men who have sex with men (…) It’s equally important to have them*,* have trans inclusion in them and trans*,* considerations of trans needs embedded in them from the start. Because there was a period of a couple of weeks where trans women I know who were high risk*,* were not getting vaccinated because they didn’t want to go into this all-male queue and potentially have to explain themselves”. (B3)*

## Discussion

Our study provides key insights into the heterogeneity of experiences of people affected by the mpox outbreak in the UK in 2022–2023 and the intersection of characteristics, practices, and circumstances that might create difficulties in accessing the mpox vaccine. All our personas represent the characteristics of someone on the margins, either geographically (by living outside of London) or socially (e.g., by not being connected to sexual health services or the LGBTQ + community). The intersection of being situated at the margin, together with other social locations, provided the potential for exclusion from the 2022 mpox vaccine campaign. These social locations included both practical ones (e.g., difficulties in accessing the vaccine) and socio-demographic-linked ones (e.g., guidance not tailored to trans women, access to NHS service for migrants, lack of trust in institutions often due to the experience of systemic racism). This occurred even though the vaccination strategy explicitly aimed “to ensure those who are not already known or registered with sexual health services are informed where and how to access the vaccine” [56]. This finding is in line with previous work that highlighted practical and logistic difficulties encountered by people seeking to get vaccinated, either due to shortages or limited availability of vaccines, resource and time constraints, lack of pre-existing connection with sexual health services, or lack of information on the centres providing vaccination [21, 23, 30–32, 34–36].

Previous studies have highlighted the essential role of sexual health services during the outbreak [14, 15], not only in the vaccination campaign but also in providing relatable and trustworthy information and creating a sense of collective responsibility, especially in a context of perceived lack of government response. However, our study and others have shown the potential for exacerbating exclusion through solely relying on existing clinical infrastructure and existing community networks – including via social media [15].

Stigma was a major theme in our study, and our equity focus also emphasizes the enhanced risk of stigma for minoritised and multiply minoritised groups. Previous studies have similarly identified stigma as a central feature of the mpox outbreak and its link to the HIV pandemic [35, 57]. The homophobic discourse in the media, and the use of stigmatising images conveying messages around the supposed sexual promiscuity of GBMSM contributed to misinformation, as GBMSM did not identify with these narratives [57]. Combined with the lack of trust in institutions, (e.g., due to previous discriminatory experience in the healthcare system) and mistrust of science in general, this had the potential for creating ambiguity towards the vaccine. Some were also questioning the balance between the benefits of the vaccine versus the potential social cost of having one’s sexuality revealed. Concerns were expressed regarding the potential of the mpox outbreak to be exploited for anti-LGBTQ + political purposes, as highlighted in other papers [18, 58, 59]. It has been suggested that clear communication by public health professionals, medical professionals and the media could not only reduce stigma but ensure broader reach to individuals on the fringes of the LGBTQ + community [59]. Additionally, the early adoption of a validated stigma scale to better understand the potential effects of stigma in a public health emergency is an important mechanism to prevent exclusion [59].

Moreover, previous research has highlighted the importance of mpox risk perception as a predictor for vaccination uptake [22, 35]. Although risk perception was mentioned by some participants, our work suggests self-perceived risk was considered alongside practical considerations and convenience related to vaccine access. The fluidity of risk, which might change along with specific circumstances (e.g., events, holidays, traveling, relationships), was not reflected in the vaccination guidelines.

This work will assist in the design of more inclusive future vaccine campaigns. Our community partners are working closely with the NHS in England and the UK Health Security Agency (UKHSA) on developing the future mpox vaccine roll-out. Our findings also have wider significance to other countries experiencing mpox outbreaks within similar population groups as our approach provides a theoretical framework for understanding potential exclusion.

Based on our findings, we thus consider that the following recommendations may support policymakers and those responsible for implementing vaccine roll-outs to address barriers inhibiting vaccine access.

1. Communication should be clear, regular and inclusive. Initial guidance around mpox risk and vaccine eligibility was criticised – both by our participants and those in other studies [30, 35, 57] – for failing to include groups beyond GBMSM who were likely to have been at elevated risk of mpox acquisition due to their sexual networks and practices, particularly trans and non-binary people. While we recognise the challenges of providing accurate information with certainty during the emergence of novel outbreaks, provision of regular and consistent updates through trusted sources which acknowledge uncertainty and build in expectations for future changes, can both help to reassure individuals and inform their risk management strategies [60, 61].
  The tension between targeted versus generalised messaging is an issue that has been long debated in other areas of sexual health, particularly HIV [62]. There is a strong evidence base to support tailored messaging to promote vaccination uptake among communities most in need or where there is low vaccine uptake [63]. However, there is clearly a need to balance this with general, mainstream messaging that informs and destigmatises. Our persona approach further highlights the need to consider whether shared barriers to engagement (which can span multiple identities and positionalities) require tailored solutions.
2. Eligibility criteria should be improved, and their rationale should be more transparent. Current mpox vaccine guidance from NHS England is still focused on GBMSM specifically, and does not include more inclusive criteria encompassing those who are not GBMSM but May have overlap in their sexual networks, including gender diverse people who do not identify with the GBMSM label. The rationale for rationing vaccines through eligibility criteria, including limited supply as was the case in 2022/23, should be clearly communicated to help maintain trust and manage expectations among communities at elevated risk.
3. Accessibility of sexual services should be improved. There are evident challenges for certain individuals in accessing sexual health services. Efforts to improve accessibility could include expanding entry points into services (e.g. telephone, online, and face-to-face), increasing provision of interpreters and translation services, and increasing appointment provision. Such changes would likely have a broader positive impact on sexual health outcomes beyond mpox vaccine uptake, e.g., in the implementation of meningococcal B vaccination for the prevention of gonorrhoea [64]. However, they will also require resourcing, against the context of considerable funding pressures for sexual health services in the UK [65]. As well as inhibiting accessibility, failure to provide additional funding for the mpox response has placed considerable pressures on sexual health staff, increasing the risk of burnout [66]. Any proposed changes to sexual health services to aid vaccine roll-out should therefore be met by additional funding. At the broader level, restoring public health funding to adequate levels and providing longer-term investment would also considerably aid outbreak prevention, preparedness and response [67] – e.g., by prioritising the needs of frontline health care workers [68].
4. The provision of vaccines should be widened beyond sexual health services, e.g., by delivering vaccination through general practitioners or pharmacies or by organising LGBTQ + events with local community organisations during which vaccination can be provided (such as what was done during Black Pride festival in London, UK). As with sexual health services, this would need to be adequately resourced.
  At all stages, it is imperative that representatives of communities most affected are involved in planning, strategy development and delivery. This includes the public health and clinical spokespeople represented by traditional media [18]. Intersectional and equity lenses can offer support in identifying which groups are not represented among existing partnerships between government and civil society. Crucially, this needs to happen in advance of emergencies arising, to build and sustain trust.

### Strengths and limitations

Thanks to our community co-produced approach, the study sample on which our personas are based had substantial demographic diversity (e.g., in terms of ethnicity and gender identity). However, participants were mostly in their thirties, which may have limited our potential to identify different perspectives from younger and older age. Previous work identified age difference in the ability to cope with mpox-related stigma [57]. Participants were recruited by The Love Tank via their existing networks and social media, meaning participants were likely more connected to health promotion than others affected by mpox. The COVID-19 pandemic and related public health measures (such as vaccines and isolation) were still ongoing when the 2022 mpox outbreak occurred; this potentially created additional barriers to mpox vaccine access which may be less relevant to future outbreaks.

The *personas* approach provides valuable insights into complex and multifaceted experiences. They allow the presentation of qualitative findings using relatable narratives that can be used beyond academic settings and inform tailored public health interventions. However, they are built as exemplary, rather than representative, cases. Therefore, they should not be read as representation of the studied populations, but as a composite of different experiences and a way to highlight some issues encountered by some people during the mpox vaccine roll-out. In other words, individuals could identify very differently from the personas described in the results, but still experience a similar barrier to vaccination due to a combination of other factors. We thus suggest the focus should be on the barriers, and how they may be relevant to lives in multiple individuals. Due to the level of detail involved, the *personas* approach can raise ethical considerations regarding anonymity of the research participants. Researchers also need to balance the risk of bias and oversimplification when constructing personas. Moreover, personas may be erroneously read as another set of identities, instead of the intended set of circumstances leading to exclusion. By following our six principles, we were able to highlight that circumstances can be shared and experienced across identities, addressing this potential limitation. The persona approach is a way to move beyond a highly identitarian way of perceiving people’s relationship with healthcare and towards a socio-material understanding [69] that recognises instead that experiences (such as barriers to access) can both disproportionately affect some people and simultaneously be shared across demographics.

## Conclusion

Our findings highlight the need to first evaluate the potential effects of stigma (e.g. using a validated tool [59]) and of different intervention approaches during an outbreak (e.g. through extensive collaboration with a variety of communities on the ground). This will assist in the design of more inclusive future vaccine campaigns during evolving public health emergencies that consider how the intersection of stigma, characteristics, practices, and circumstances might contribute to exclusion from vaccine provision and inhibit uptake. Any guidelines should be co-designed with a range of affected communities and include stigma-reduction approaches to ensure equitable provision and access to vaccine programmes. For mpox specifically, it remains critical to consider how barriers presented in this study may influence access to and uptake of mpox vaccines in ongoing outbreaks in very different geographical contexts. The method suggested here could be replicated through engagement with appropriate community representatives in different settings to identify barriers and develop tailored interventions [70].

## Supplementary Information

Supplementary Material 1.

## Data Availability

The data from this study will not be shared due to the sensitive nature of the topics discussed and the risk of re-identification despite anonymisation.

## Abbreviations

WHO: World Health Organization
MPXV: Mpox virus
GBMSM: Gay, bisexual, and other men who have sex with men
STI: Sexually transmissible infection
HIV: Human immunodeficiency virus
ADHD: Attention-Deficit/Hyperactivity Disorder
NHS: National Health Service
UKHSA: UK Health Security Agency
