# Supplementary material for: ‘It was something that I let slip by the wayside’: a qualitative study of challenges to equitable mpox vaccination in the UK outbreak response
Source: BMC Public Health. 2025 Oct 31;25:3678. doi: 10.1186/s12889-025-24694-w (PMC12577179; doi:10.1186/s12889-025-24694-w)
Supplement: Supplementary file 1 — Supplementary Material 1. [file 12889_2025_24694_MOESM1_ESM.pdf]

Supplementary material for '*It was something that I let slip by the wayside*': a qualitative study of challenges to equitable mpox vaccination in the UK outbreak response
Pasin, Weil, et al

Supplementary material 1

PARTICIPANT INTERVIEW TOPIC GUIDE

Study title: Exploring knowledge and experience of Mpox harm reduction interventions amongst most affected communities in the United Kingdom

Demographic information:

Gender	
Sex at birth	
Age	
Ethnicity	
Sexual orientation	
Ability	
Country of birth	
Employment status	
Occupation	
Education level achieved	

TOPIC GUIDE FOR INDIVIDUAL SEMI-STRUCTURED INTERVIEW

Note to interviewer: Depending on the flow of conversation during the interview, the participant may answer questions contained within the topic guide that you haven't yet asked. That is fine! Don't feel constrained by the order of the questions below, but please do ensure all questions have been covered by the end of the interview.

Topic	Question rationale	Questions	Potential prompts
Introduction	To establish rapport	How are you doing?	*Can you tell me a bit about yourself?*
Experiences of MPOX health promotion	To introduce the interview topic	When did you first hear about MPOX?	*Can you tell me a bit about that time?*
	To understand exposure to MPOX health	Do you remember seeing any health guidance or	• *In the news*<br>• *On social media*<br>• *At the sexual health clinic*

	promotion material	information about MPOX?	• *At LGBT community venues*<br>• *Government website*
	To understand perceptions of MPOX health promotion material	**If yes:** How did you feel about it?	*Did you think it was helpful?*<br>*Did you think it was relevant for you and/or people like you?*<br>*(Note: could be different for different kinds of sources)*
Communicating about MPOX	To understand whether the participant communicated with others about MPOX	Did you talk to others about MPOX around that time	• *Friends*<br>• *Partners*<br>• *Family*<br>• *Healthcare professionals*<br>• *Others in the community*
	To understand what information about MPOX was shared	**If yes:** What did you talk about?	*Did you have specific questions?*<br>*Did you have concerns?*<br>*For yourself or for others you know?*
	To understand barriers to communicating about MPOX	**If no:** Why not?	*Did you feel you had insufficient information?*<br>*Did you feel awkward discussing it?*<br>*Did you feel others would not know much about it?*
Behaviour change to prevent MPOX acquisition	To understand if and what type of behaviour change occurred to prevent MPOX acquisition	Did you do anything differently to try and avoid getting MPOX around that time?	• *Reducing number of sexual partners*<br>• *Doing different things with different people*<br>• *Changing social environments*<br>• *Avoiding specific social spaces*<br>• *Using condoms*<br>• *Change in types of sexual contact/activity*<br>• *Stopping sex altogether*<br>• *Evaluating sexual partners (e.g., asking about symptoms, checking for lesions)*

	To understand individuals' perception of their own risk of acquiring MPOX	Can you tell me what your reasons were for doing or not doing) things differently?<br><br>(Note: respondents may discuss behaviour change due to having MPOX – if this is the case, see questions below on the topic 'Behaviour change to prevent onward MPOX transmission'.	• *Didn't think they were at risk because of sexual activity*<br>• *Didn't thinks they were at risk because of sexual partners*<br>• *Didn't think they were at risk because they had received a vaccine/ had immunity*<br>• *Thought they were at risk because.....*<br>• *Didn't want to risk transmission to others (partners, family, friends, workplace)*
	To understand duration of behaviour change	**If they changed behaviour:** How long would you say you did that for?	*A one off, a few days, weeks, or months?*
	To understand sustainability and experience of change	**How was this for you?**	• *Easy to do*<br>• *Easy to be consistent*<br>• *Hard to be consistent*<br>• *Hard to do*
	To understand if the participant observed behaviour change to prevent MPOX acquisition among others	Did you notice anyone else doing anything differently to try and avoid getting MPOX around that time?	*What kind of things were they doing differently?*
Potential immunity prior to MPOX outbreak	To understand if there was any possible MPOX exposure prior to the outbreak	Do you remember having any [list symptoms of MPOX] in the weeks or months before the MPOX outbreak was announced in May 2022?	
	To understand whether treatment was sought and diagnostic / treatment outcomes	How did you deal with these?	*Did you ask for help at a pharmacy, GP or sexual health clinic?*<br>*What happened?*

Experiences of MPOX treatment	To understand experience of MPOX infection	Do you think you got MPOX at any point during the outbreak?	*Why do you think?*<br>*When did this happen?*<br>*Did you get tested?*
	To understand experience of MPOX treatment	**If yes:** Did you access treatment for MPOX?	*Did you go to the GP?*<br>*Or sexual health clinic?*<br>*Or A&E?*<br>*Or pharmacy?*<br>*Or community org?*
	To understand access to MPOX treatment	**If yes:** How was your experience of accessing treatment?	*Did you feel confident in the care received?*<br>*Did it feel straightforward to receive care?*<br>*Did you get consistent response from healthcare professionals?*<br>*Did you feel you had to advocate for yourself or was it easy?*
		**If no:** Why didn't you access treatment?	*Was there anything that put you off accessing treatment?*<br>*Was there anything you'd have liked to have happen differently?*
Behaviour change to prevent onward MPOX transmission	To understand if and what type of behaviour change occurred to prevent onward MPOX transmission	Did you do anything differently in relation to your sexual life while you had MPOX symptoms?<br><br>What about your social life?	• *Abstinence for a given period of time*<br>• *Not going on dates in the first place*<br>• *Reduction of number of sexual partners*<br>• *Reduction of social life/events*<br>• *Condom use*<br>• *Change in types of sexual contact/activity*<br>• *Disclosure to partners*<br>• *Covering of lesions*
	To understand participation in MPOX harm reduction interventions?	Can you tell me what your reasons were for doing (or not doing) this?	• *Following guidance*<br>• *Not being clear about transmission risk*<br>• *Worried about passing on to others*<br>• *Taking responsibility for others in the community*

			• *Couldn't take time off work*<br>• *Fear of others finding out about their sexuality*<br>• *Concerns about stigma*
	To understand duration of behaviour change to prevent onward MPOX transmission	**If they changed behaviour:** For how long did you change your behaviour?<br><br>Why this long?	*A few days, weeks, or months?*
	To understand sustainability and experience of change	**How was this for you?**	*Easy to do*<br>*Easy to be consistent*<br>*Hard to be consistent*<br>*Hard to do*
Testing for asymptomatic MPOX infection among people at increased risk of MPOX	To understand acceptability of asymptomatic testing	If tests were available for people who didn't have MPOX symptoms, would you like to test for MPOX as part of a sexual health check-up, even if you didn't have any symptoms? This might be something like a swab in the throat or rectum.	*For example, say you wanted to get a STI check-up, would you like to be tested for MPOX too?*
		Can you tell me why? Or why not?	• *Worried about having asymptomatic MPOX*<br>• *Wanting to be sure of safe sex for them and others*<br>• *Stigma*<br>• *Don't think they are at risk*<br>• *Have been vaccinated (how many times?)*
	To understand preferences for asymptomatic testing setting	**If yes:** Where would you want to access an MPOX test?<br><br>Why in this location?	• *Sexual health clinic*<br>• *GP*<br>• *Pharmacy*<br>• *Community organisation*

			• *Self-sampling at home*
Ending		Is there anything else you'd like to add or to ask?<br><br>THANK YOU VERY MUCH FOR YOUR TIME	

Supplementary material for '*It was something that I let slip by the wayside*': a qualitative study of challenges to equitable mpox vaccination in the UK outbreak response
Pasin, Weil, et al

Supplementary material 2

FGD TOPIC GUIDE

Study title: Exploring knowledge and experience of Mpox harm reduction interventions amongst most affected communities in the United Kingdom

Demographic information:

Gender	
Sex at birth	
Age	
Ethnicity	
Sexual orientation	
Ability	
Country of birth	
Employment status	
Occupation	
Education level achieved	

TOPIC GUIDE FOR FOCUS GROUP DISCUSSION

- Welcome and moderator introduction
- Overview of the study topic / why they've been asked to attend
- Ground rules
- First question

Topic	Question rationale	Questions	Potential prompts
Introduction	To establish rapport	[Round robin to introduce everyone prior to recording]	
Experiences of MPOX health promotion	To introduce the interview topic	When did you first hear about MPOX?	*Can you tell me a bit about that time?*
	To understand exposure to MPOX health promotion material	Do you remember seeing any health guidance or information about MPOX?	• *In the news*<br>• *On social media*<br>• *At the sexual health clinic*<br>• *At LGBT community venues*

			• *Government website*
	To understand perceptions of MPOX health promotion material	How did you feel about it?	*Did you think it was helpful?*<br>*Did you think it was relevant for you and/or people like you?*<br>*(Note: could be different for different kinds of sources)*
Communicating about MPOX	To understand whether the participant communicated with others about MPOX	Did you talk to others about MPOX around that time?	• *Friends*<br>• *Partners*<br>• *Family*<br>• *Healthcare professionals*<br>• *Others in the community*
	To understand what information about MPOX was shared	**If yes:** What did you talk about?	*Did you have specific questions?*<br>*Did you have concerns? For yourself or for others you know?*
	To understand barriers to communicating about MPOX	**If no:** Why not?	*Did you feel you had insufficient information?*<br>*Did you feel awkward discussing it?*<br>*Did you feel others would not know much about it?*
Behaviour change to prevent MPOX acquisition	To understand if and what type of behaviour change occurred to prevent MPOX acquisition	There are a range of behaviour changes that could help to reduce your chances of acquiring mpox during an outbreak (*show the list*).<br><br>How did you feel about these options?<br><br>Did you take any of them up? Was it easy or difficult?<br><br>Is there anything on this list that you think would be impossible to do?	• *Reducing number of sexual partners*<br>• *Doing different things with different people*<br>• *Changing social environments*<br>• *Avoiding specific social spaces*<br>• *Using condoms*<br>• *Change in types of sexual contact/activity*<br>• *Stopping sex altogether*<br>• *Evaluating sexual partners (e.g., asking about symptoms, checking for lesions)*

	To understand duration of behaviour change	**If they changed behaviour:** How long would you say you did that for?	• *A one off, a few days, weeks, or months?*
	To understand individuals' perception of their own risk of acquiring MPOX	Why do you think people made or didn't make these behaviour changes?	• *Didn't think they were at risk because of sexual activity*<br>• *Didn't think they were at risk because of sexual partners*<br>• *Didn't think they were at risk because they had received a vaccine/ had immunity*<br>• *Thought they were at risk because.....*<br>• *Didn't want to risk transmission to others (partners, family, friends, workplace)*
Behaviour change to prevent onward MPOX transmission	To understand if and what type of behaviour change occurred to prevent onward MPOX transmission	If you have mpox infection, there are a range of behaviour changes that could help to reduce your chances of passing mpox on to someone else.<br><br>How do you feel about these options?<br><br>Do you think they would be easy or hard to engage in?	• *Abstinence for a given period of time*<br>• *Not going on dates in the first place*<br>• *Reduction of number of sexual partners*<br>• *Reduction of social life/events*<br>• *Condom use*<br>• *Change in types of sexual contact/activity*<br>• *Disclosure to partners*<br>• *Covering of lesions*
	To understand participation in MPOX harm reduction interventions?	Why do you think people made or didn't make these behaviour changes?	• *Following guidance*<br>• *Not being clear about transmission risk*<br>• *Worried about passing on to others*<br>• *Taking responsibility for others in the community*<br>• *Couldn't take time off work*

			• *Fear of others finding out about their sexuality*<br>• *Concerns about stigma*
Testing for asymptomatic MPOX infection among people at increased risk of MPOX	To understand acceptability of asymptomatic testing	If tests were available for people who didn't have MPOX symptoms, would you like to test for MPOX as part of a sexual health check-up, even if you didn't have any symptoms? This might be something like a swab in the throat or rectum.	*For example, say you wanted to get a STI check-up, would you like to be tested for MPOX too?*
		Can you tell me why? Or why not?	• *Worried about having asymptomatic MPOX*<br>• *Wanting to be sure of safe sex for them and others*<br>• *Stigma*<br>• *Don't think they are at risk*<br>• *Have been vaccinated (how many times?)*
	To understand preferences for asymptomatic testing setting	**If yes:** Where would you want to access an MPOX test?<br><br>Why in this location?	• *Sexual health clinic*<br>• *GP*<br>• *Pharmacy*<br>• *Community organisation*<br>• *Self-sampling at home*
Ending		Is there anything else you'd like to add or to ask?<br><br>THANK YOU VERY MUCH FOR YOUR TIME	
